# Common gene-network signature of different neurological disorders and their potential implications to neuroAIDS

**DOI:** 10.1371/journal.pone.0181642

**Published:** 2017-08-08

**Authors:** Vidya Sagar, S. Pilakka-Kanthikeel, Paola C. Martinez, V. S. R. Atluri, M. Nair

**Affiliations:** Institute of Neuroimmune Pharmacology/Center for Personalized Nanomedicine, Department of Immunology, Herbert Wertheim College of Medicine, Florida International University, Miami, Florida, United States of America; Rutgers University, UNITED STATES

## Abstract

The neurological complications of AIDS (neuroAIDS) during the infection of human immunodeficiency virus (HIV) are symptomized by non-specific, multifaceted neurological conditions and therefore, defining a specific diagnosis/treatment mechanism(s) for this neuro-complexity at the molecular level remains elusive. Using an in silico based integrated gene network analysis we discovered that HIV infection shares convergent gene networks with each of twelve neurological disorders selected in this study. Importantly, a common gene network was identified among HIV infection, Alzheimer’s disease, Parkinson’s disease, multiple sclerosis, and age macular degeneration. An mRNA microarray analysis in HIV-infected monocytes showed significant changes in the expression of several genes of this in silico derived common pathway which suggests the possible physiological relevance of this gene-circuit in driving neuroAIDS condition. Further, this unique gene network was compared with another in silico derived novel, convergent gene network which is shared by seven major neurological disorders (Alzheimer’s disease, Parkinson’s disease, Multiple Sclerosis, Age Macular Degeneration, Amyotrophic Lateral Sclerosis, Vascular Dementia, and Restless Leg Syndrome). These networks differed in their gene circuits; however, in large, they involved innate immunity signaling pathways, which suggests commonalities in the immunological basis of different neuropathogenesis. The common gene circuits reported here can provide a prospective platform to understand how gene-circuits belonging to other neuro-disorders may be convoluted during real-time neuroAIDS condition and it may elucidate the underlying–and so far unknown–genetic overlap between HIV infection and neuroAIDS risk. Also, it may lead to a new paradigm in understanding disease progression, identifying biomarkers, and developing therapies.

## Introduction

The clinicopathological characteristics of different neurological disorders are often indistinguishable. Symptoms such as hallucinations, memory loss, dementia, and other psychotic symptoms are common to almost all neuro-disorders. Accordingly, the existing “partial-treatment” strategy, which primarily includes several decades old prototype drugs, are meant to antagonize targets which are common for most neuro-disorders (e.g. D2 dopamine receptors antagonists) [1,2]. Improvement towards disease-specific targets will require sincere insight into their complex molecular mechanisms, which has started only recently with the advent of high-throughput genomic technologies. A correlation at the interface of neurobiology and human genetics is being drawn with the discovery of multiple genetic signatures belonging to a single disease or common to various neuro-disorders. Disrupted expression of even a single gene possesses the ability to affect several molecular pathways and therefore, multiple psychotic phenotypes may have connection in converged molecular circuits. Nonetheless, analysis of underlying disease processes based on a single gene annotation is less comprehensive[3]. Thus, the discovery of shared dysregulated molecular pathways among various neuro-disorders can accelerate mechanistic studies of their pathophysiology which, in turn, can lead to appropriate therapies.

Neuroinvasion by human immunodeficiency virus (HIV) cause several types of neurologic conditions which are collectively termed as the “neuroAIDS” [4–7]. The presence of HIV particles/proteins/genetic components and intrathecal anti-HIV antibodies in the brain and CNS are detected early during infections. This suggests that HIV enters the brain during early infection phase and therefore it is believed that the neuroAIDS condition in HIV patients can prevail throughout the infection period [6,8–10]. Initial entry of HIV into the brain is mediated via infected monocytes and macrophages from the peripheral circulation. These mononuclear phagocytes (irrespective of being non-infected and/or infected) intrinsically possess specific cytokines/chemokines-responsive, transendothelial migration ability to cross the blood-brain barrier (BBB) and go into the brain (e.g. monocyte chemotactic protein-1) [11–13]. Early HIV infection of brain cells triggers the release of BBB-compromising factors (e.g. matrix metalloproteinase) [14] which induces the influx rate of inflammation responsive leukocytes from peripheral body region into the brain [15]. Now, both infected and non-infected monocytes/macrophages from peripheral circulation reach to the brain in higher number than the normal condition [16]. Higher accumulation of infected leukocytes intensifies the progression of HIV infection in different brain cells. Among the major brain cells, the susceptibility of astrocytes and microglia to HIV infection has been shown in various studies [17]. However, HIV invasion to nerve cells remains highly debated. Nonetheless, neurotoxic effect of HIV proteins on neurons is a proven phenomenon [18]. HIV infections to different cell types are not always productive; rather, HIV acquires latency in a subpopulation of almost all cell types including brain cells [19–21]. Latency allows HIV to escape deleterious effects of cellular immune response and/or antiretroviral drugs. Transcription of host-integrated HIV genome during the latency phase is zero or minimum. Latency can persist for years and it can lead to chronic pathological implications because minimum viral genome transcription can continuously produce little virus and/or viremia can be rebound upon latency reactivation [22]. In fact, HIV reactivation in a fraction of latent cells due to specific endogenous or exogenous stimulus is a continuous occurrence in a real physiological condition which adds to the disease progression[6,20]. Thus, both latent, as well as active HIV infections, contribute to the neuroAIDS condition.

The health and function of brain regions and spinal cord involved in learning and information processing are significantly compromised due to the neuroAIDS condition. While nearly 50% HIV patients demonstrate one or other kind of neurological symptoms [23], a much higher percentage of autopsies of AIDS patients (~80%) shows mild to severe neurological deformities [24]. The neuroAIDS is shaping up as a global problem and this can be attributed to: (i) longevity of HIV patients upon consistent HAART treatment, (ii) ineffectiveness of HAART for HIV-associated neurological complications, (iii) lack of ARV therapies for ~ 40% of AIDS patients, (iv) existing non-specific, common “partial-treatment” strategy for most neuro-disorders, and (v) inability of more than 98% drugs to transmigrate across the blood-brain barrier (BBB) [6]. Some notable neurological complications within the spectrum of neuroAIDS are HIV-associated neurocognitive disorder (HAND), chronic meningitis, peripheral neuropathies, neurosyphilis, CNS lymphomas, progressive multifocal leukoencephalopathy, vacuolar myelopathy, etc. Also, neurological deformities of unknown origin are seen in HIV patients [4,5,7]. To date, the precise diagnosis of neuroAIDS onset remains a formidable task for scientific communities because of its intrinsic multifaceted symptoms and pathologies.

Neurodegeneration by HIV virion and neurotoxic HIV proteins involve complex etiology. Differentiation of HIV-infected monocytes into macrophages leads to astrocytes and microglia activation which cause severe neuroinflammation. The subsequent release of neuron-damaging molecules such as reactive oxygen species, nitric oxide, TNF-α, IL-1β, quinolinic acid, β-chemokines, arachidonic acid, etc. further exacerbates the pathological process [17,25,26]. The release of these proinflammatory factors in macrophages and microglia is elevated by HIV envelope protein gp120. The gp120 activates chemokine receptors (CXCR4 or CCR5) in neurons which induce rise of intracellular Ca^2+^ concentration leading to apoptosis [27]. In astrocytes, gp120 induces excitotoxicity and cell death via downregulating glutamate uptake and increasing production of nitric oxide synthase production, respectively [28,29]. The gp120 protein also inhibits neural progenitor cells (NPCs) migration and proliferation [30]. Additionally, gp120 induces apoptosis in the brain microvascular endothelial cells (BMVECs) to alter BBB integrity [31]. Activation of apoptosis by gp120/gp41, especially via the p53 pathway, is a common phenomenon across all three major brain cells i.e. macrophages/microglia, neurons, and astrocytes [25]. The HIV Tat protein stimulates hostile conditions for neurons and other brains cells in many ways which are similar to gp120. Exposure of Tat elevates the proinflammatory factors such as TNF-α and IP-10 in macrophages and microglia, induces apoptosis in BMVECs, upregulates MCP-1 and reduces glutamate uptake in astrocytes, and inhibits NPCs neurogenesis [25,32]. In neurons, Tat induces NMDA receptors, activate NO and Ca-release, inhibits tyrosine hydroxylase, and decrease dopamine [25,32]. These effects of Tat proteins lead to neuronal death by apoptosis or other cytotoxicity means. The HIV Vpr and Nef proteins also induce apoptosis in BMVECs, astrocytes, and neurons. In addition, Vpr exposure modulates ion channels and H_2_O_2_ upregulation in neurons, impairs neuron maturation, and induces mitochondrial dysfunction in NPCs [17,33]. Exposure of Nef protein elevates proinflammatory factors (TNF-α, MIP-1, IL-6, etc.) and superoxide release in macrophages and microglia [34,35]. In neurons, Nef modulates [K^+^] ion channels and in astrocytes, it upregulates complement factor C3, MCP-1, IP-10, and MMP-9 activity [17,25,26]. While all these mechanisms certainly aggravate the neuroAIDS pathologies in one or other way, HIV infection also leads to synaptodendritic injury. Neuronal spine density, dendritic diameter, total spine, and dendritic area are significantly compromised during the HIV infection [36–38]. This may be correlated with the atrophy of grey and white matter in the brain of HIV patients [39]. Many of similar molecular processes involving apoptosis, generation of reactive oxygen species, generation of proinflammatory factors, ionic channel modulations, synaptodendritic injury, etc. are seen in the case of Alzheimer’s disease [40], Parkinson’s disease [41], multiple sclerosis [42], age macular degeneration [43] or other neurological disease. In fact, gene expression changes during neuroAIDS condition show consistency with these neuro-disorders. Such as, HIV infection induces production of amyloid precursor protein and subsequently amyloid is accumulated in the brain [44]. Similarly, IGF signaling has been implicated in both neuroAIDS and PD; and ErbB2/B4 and NRG1, responsible for schizophrenia susceptibility, are found in neuroAIDS as well [45]. Thus multiple gene-circuit crossovers between neuroAIDS and other neuro-disorders are suspected. Nonetheless, barring some sporadic reports with few genes related to a specific disease; studies comparing gene-network signatures between HIV infection and neuro-disorders are lacking in entirety. As such discovering these molecular crossovers may reveal the reason for multifaceted symptoms and pathologies of the neuroAIDS condition.

An intensive bioinformatics analysis was performed between and among the molecular networks of risk-associated genetic markers for HIV infection and twelve major neurological disorders (Alzheimer’s disease, Parkinson’s disease, Multiple Sclerosis, Age Macular Degeneration, Amyotrophic Lateral Sclerosis, Vascular Dementia, Restless Leg Syndrome, Glaucoma, Migraine, Creutzfeldt—Jakob disease, Narcolepsy, and Autism-Autism Spectrum) obtained from the genome-wide association study (GWAS) catalog. The bioinformatically discovered common gene circuits in this study, in combination with mRNA microarray for HIV latent and active infection model, suggest similarity in the pattern of genetic disruption for HIV infection and neuro-disorders. As such, common gene-network signatures reported here can have significant physiological relevance in driving convoluted nature of pathologies during neuroAIDS and other neurologic conditions.

## Materials and methods

### Bioinformatics analysis

All established at-risk alleles of HIV infection and twelve major neurological disorders (Alzheimer’s disease, Parkinson’s disease, Multiple Sclerosis, Age Macular Degeneration, Amyotrophic Lateral Sclerosis, Vascular Dementia, Restless Leg Syndrome, Glaucoma, Migraine, Creutzfeldt—Jakob disease, Narcolepsy, and Autism-Autism Spectrum) delineated in Genome-Wide Association Studies (GWAS) online catalog were retrieved and were denoted as “at-risk” genes (S4 Data). These genome-disease associations listed in GWAS are based on different population-based studies across the globe where expression modulations and polymorphism in one and/or other genome have been shown to drive a specific disease in respective populations. One or more than one GWAS genes from a single study for a disease in one population may not be primary reason of the same disease in other population from another study. In many cases, the primary gene responsible for a disease in one population can be secondary or tertiary responsible gene or can be far-related as well in other population. Thus at-risk genes for a disease in GWAS are an accumulation of all reported genes for that disease from different study across the globe. Gene networks of risk-associated genes of each disease were obtained in silico (bioinformatically) using the Cytoscape open software bioinformatics program and two of its plugins, GPEC v2.8.3 (Gene Prioritization and biomedical Evidence Collection) and Genemania v3.3.1 [46]. At-risk genes were run through GPEC to identify gene network associated with specific diseases through a random walk algorithm with restart. The random walk algorithm with restart is a gene prioritization method where starting nodes are selected in a given network and subsequently with a certain probability value, random walker either moves to a random immediate neighbor node from the starting node or returns to one of the starting nodes [47]. Specifications for GPEC run are: Step 1: The network identified is the Default Human Functional Linkage (FLN), the identifier is Entrez Gene ID and the network weighed option is selected. Step 2: The other genes/proteins option is selected, and the identifier for this step is Official Symbol. The “check” option is selected, and all the at-risk genes are added to the list. Step 3: The candidate set is selected to be neighboring interactants of at-risk genes in the network with a neighboring distance of 1. Using a random walk with the algorithm, GPEC now finds any neighboring interactants of at-risk genes with a topological distance of 1. The genes in the developed network are now known as the “neighboring genes”. Step 4: Each neighboring set is then scored and ranked by a parameter of α = 0.5. The neighboring genes networks of each neurological disorder were individually venn-analyzed in comparison with that for HIV infection. Genes common to both diseases were manually verified and selected for functional analysis performed by the second Cytoscape plugin, Genemania v3.3.1. For each particular disease, the at-risk genes of that disease were inputted along with the at-risk genes of HIV infection and the genes shared between that specific neurological disease and HIV infection. The generated visual networks of common genes by Genemania v3.3.1 were further analyzed for their involvement in various molecular pathways. The q-value for these networks indicates the probability that appears significant due to random chance [48]. Eventually, different neighboring interactants were compared together to obtain a set of common genes which were used to generate common gene-network signatures using Genemania v3.3.1.

### Microarray analysis

Unstimulated promonocytic U1 latent cells (AIDS Research and Reference Reagent Program, NIAID, National Institute of Health, Rockville, MD) were used to perform latent infection model [49,50]. Stimulation of U1 cells by PMA (10nM) for 4 hr and subsequent culture for 5 days was exerted for confirmation of latent condition (S1 Fig). The U-937 monocytes (American Type Culture Collection) infected with HIV-1_Ba-L_ (NIH AIDS Research and Reference Reagent Program catalog no. 510) were used for the active infection model. In brief, after 4 hr PMA (10nM) treatment (to maintain equivalent treatment condition as that of U1) followed by 2 hr HIV treatment (@ 6.2ngml^−1^) cells were washed with PBS buffer and returned to culture for 5 days. In other words, a set of uninfected U937 cells and stimulated U1 cells were used as controls. Experiments were performed three times (N = 3) with two technical replicates in each case. After 5 days, supernatants from both active and latent cell culture were quantified for p24 antigen (N = 3) via ELISA kit (ZeptoMetrix, Buffalo, NY) (S2 Fig). Parallely, RNAs from cells were isolated using Illustra triplePrep Kit (GE Healthcare Life Sciences, UK) and were sent to Arraystar Inc., MD, USA for mRNA microarray analysis (N = 2). In brief, purified mRNA (mRNA-ONLY™ Eukaryotic mRNA Isolation Kit, Epicentre) was amplified and subsequently transcribed into fluorescent cRNA utilizing a mixture of oligo(dT) and random primers (Arraystar Flash RNA Labeling Kit, Arraystar). The GeneSpring GX v12.1 software package (Agilent Technologies) was used for quantile normalization and subsequent data processing. The mRNAs that at least four out of twelve samples have flags in present or marginal (“All Targets Value—mRNAs”) were selected for the screening of differentially expressed mRNAs. Differential expression was considered for mRNAs with statistical significance that passed Volcano Plot filtering (Fold Change > = 2.0 and P-value < = 0.05).

## Results

### Shared gene network between HIV/AIDS and neurological disorders

**Fig 1:**
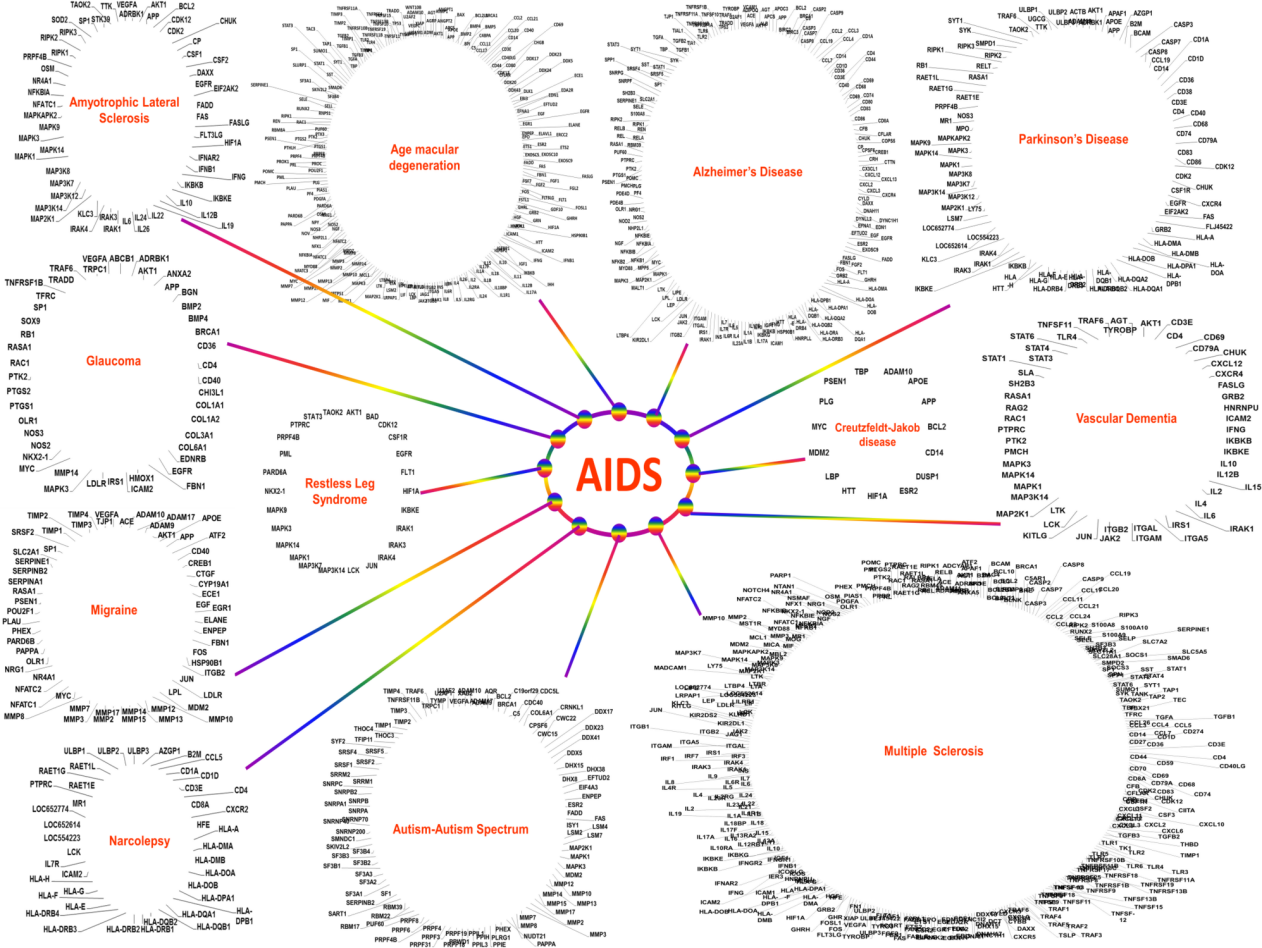
AIDS share common molecular pathways (genes) with different neurological disorders (please see S2 Data for details): neighboring gene network of each neurological disorder was compared (Venn Analysis) with that of AIDS to obtain their shared genes. All at-risk genes (S4 Data) for Schizophrenia, major depression, Bipolar and Attention Deficit Hyperactivity Disorder overlapped with Autism spectrum and as such was not classified as a separate disease.

To examine the molecular linkage between HIV infection and different neurological disorders, we utilized a “random walk algorithm with restart” within the human functional linkage network using the Cytoscape open software bioinformatics program. Functional linkage network of each disease was derived using at-risk genes retrieved from the GWAS catalog. A total of 9 genetic loci (at-risk genes) associated with AIDS risk was found which were used for network generation using Gene Prioritization and Evidence Collection (GPEC) plug-in of Cytoscape-2.8.3. With a topological distance of ≤1, GPEC could find 785 neighboring genes in the interactants of these at-risk genes. Similarly, neighboring genes of other disease were obtained (S1 Table). Venn analysis of HIV infection interactants with other disease revealed shared neighboring genes between HIV infection and each of 12 neurological disorders selected in this study (Fig 1 & S1 Data). Multiple sclerosis showed the highest number of shared genes with AIDS, followed by age macular degeneration, Alzheimer’s disease, autism-autism spectrum, and so on. The least number of shared genes were found between HIV infection and Creutzfeldt—Jakob disease (S1 Table). Visual networks of at-risk and shared neighboring genes of AIDS and each neuro-disorder were created using Cytoscape’s geneMANIA plug-in, which revealed the involvement of several important functional pathways (S2 Data). In-large, pathways related to immune-response was seen in all visual networks.

### The physiological relevance of *in silico* discovered common gene-network signatures for AIDS and neurological disorders during latent and active HIV infections

**Fig 2:**
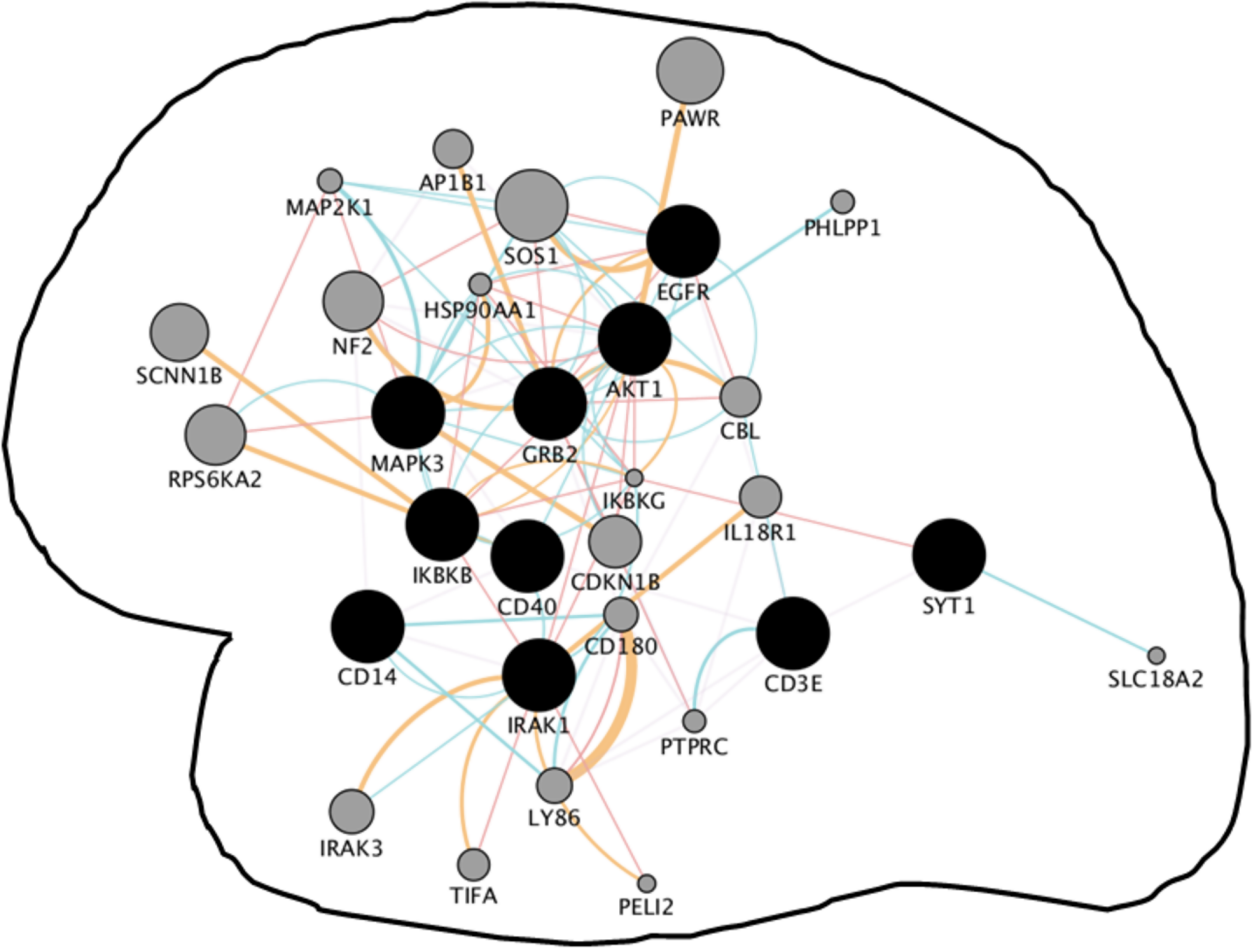
Convergent neighboring gene network shared by AIDS, Alzheimer’s disease, Parkinson’s disease, multiple sclerosis, and age macular degeneration: Convergent gene networks are primarily the shared molecular pathways between networks of genes of two or more different diseases. In our case, the term neighboring is added because networks of individual diseases represent neighboring interactants of at-risk genes. The developed network revealed the involvement of immunological pathways as retrieved by geneMANIA with P< 10^−10^. The network was developed using the 10 common genes (black dots) found via Venn analysis. Few of these common genes are also seen in other neuro-disorders: Amyotrophic Lateral Sclerosis (AKT1, EGFR, & IKBKB), Glaucoma (AKT1 & EGFR), Vascular Dementia (AKT1 & IKBKB), and Migraine and Restless leg syndrome (AKT1). Narcolepsy, Creutzfeldt-Jakob disease, and Autism-autism spectrum did not share genes with these common gene sets suggesting least chance of these symptoms during neuroAIDS. Genes in grey dots represent networking genes retrieved by geneMANIA where smaller to higher grey circle sizes represents weaker to the stronger degree of connectivity/associations between two proteins. Colored connecting line between two genes/proteins indicates interactions between them (red-physical; orange-predicated, and green-genetic) and blue lines are part of pathways. Pathways involved in this gene circuit, together with the pathways involved in Fig 4, suggests commonalities in the immunological basis of different neuropathogenesis.

**Fig 3:**
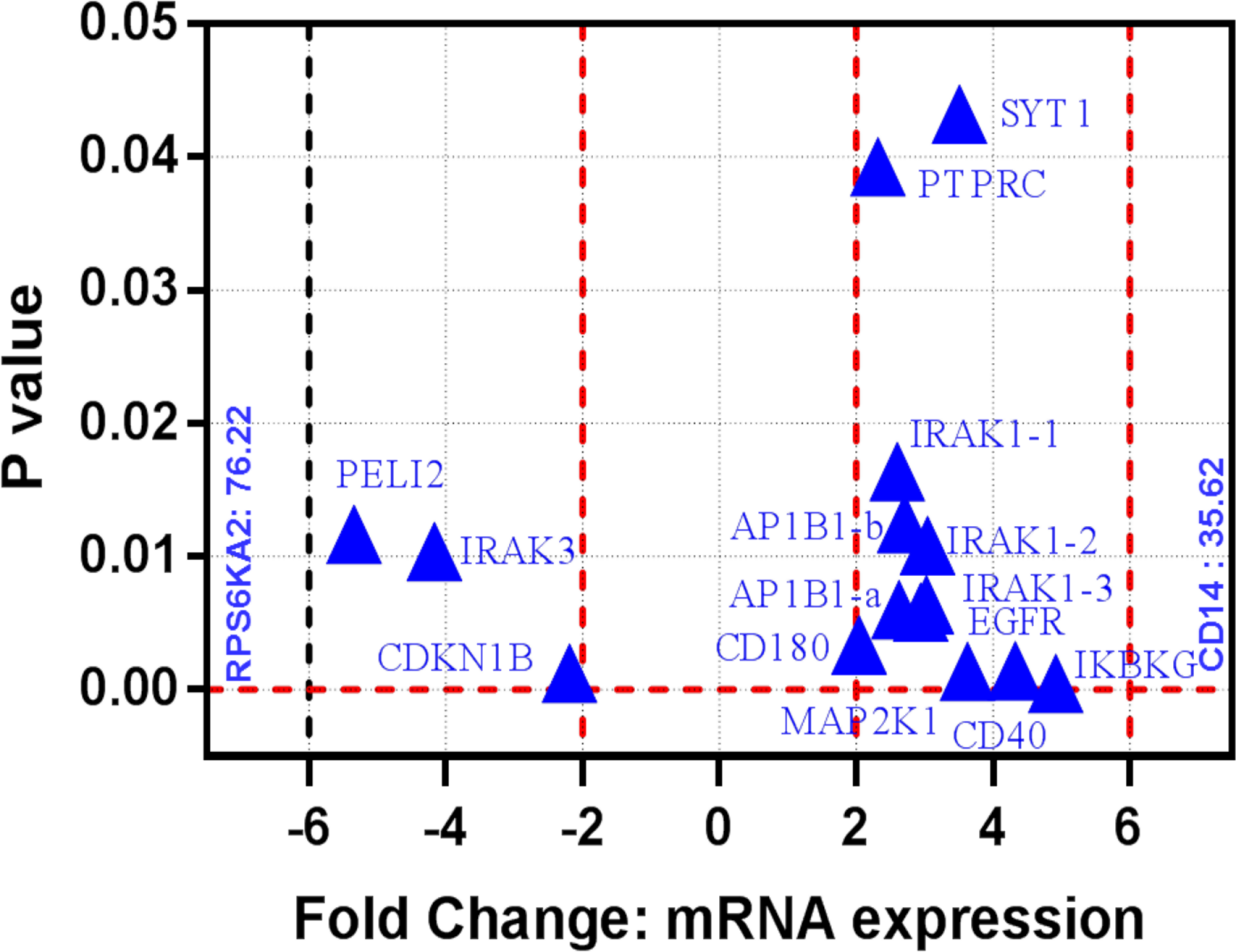
Volcano plots of the unpaired t-tests of the mRNA expression fold change for active vs latent HIV infections in monocytes (N = 2): Volcano plot is primarily a scatter plot that is used for identification of changes in specific data sets by plotting significance value versus fold-change on the y- and x-axis respectively. Our study show differentially expressed mRNAs with statistical significance that passed Volcano Plot filtering (Fold Change > = 2.0 and P-value < = 0.05). Most mRNAs showed variations with 2 to 6 fold; nonetheless, CD14 and RPS6KA2 mRNAs were 35.62 and 76.22 fold upregulated and downregulated, respectively. The vertical lines correspond to 2.0-fold up and down and the horizontal line represents a P-value up to 0.05. So the blue triangles in the plot represent the differentially expressed mRNAs with statistical significance. This represents subsets of differentially expressed mRNAs from common convergent neighboring gene network shared by AIDS, Alzheimer’s disease, Parkinson’s disease, multiple sclerosis, and age macular degeneration.

Shared neighboring interactants of HIV infection and neurological disorders were venn-analyzed and subsequently manually verified to define common gene-network signatures. We discovered 10 common genes among HIV infection, Alzheimer’s disease, Parkinson’s disease, multiple sclerosis, and age macular degeneration (S3 Data; Fig 2). Visual networks of these common genes were created using Cytoscape’s geneMANIA plug-in, which involved a total of 30 genes. Functional analysis of this gene cluster showed their involvement in pathways associated with immune response-activating signal transduction, toll-like receptor signaling, activation of immune response pattern recognition receptor signaling, MyD88-dependent signaling, etc. Further, the physiological relevance of this common network was established in the active and latent HIV infection model. For this purpose, mRNA microarray analysis was performed in the monocytes with active and latent HIV infection. It was found that > 55% genes of *in silico* derived common gene network are either upregulated or downregulated in active vs latent HIV infections. While most of these genes showed positive or negative dysregulation between 2 to 6 fold, a couple of them showed much higher fold-differences in active vs latent infection (Fig 3).

### Converged molecular circuits of major neurological disorders

**Fig 4:**
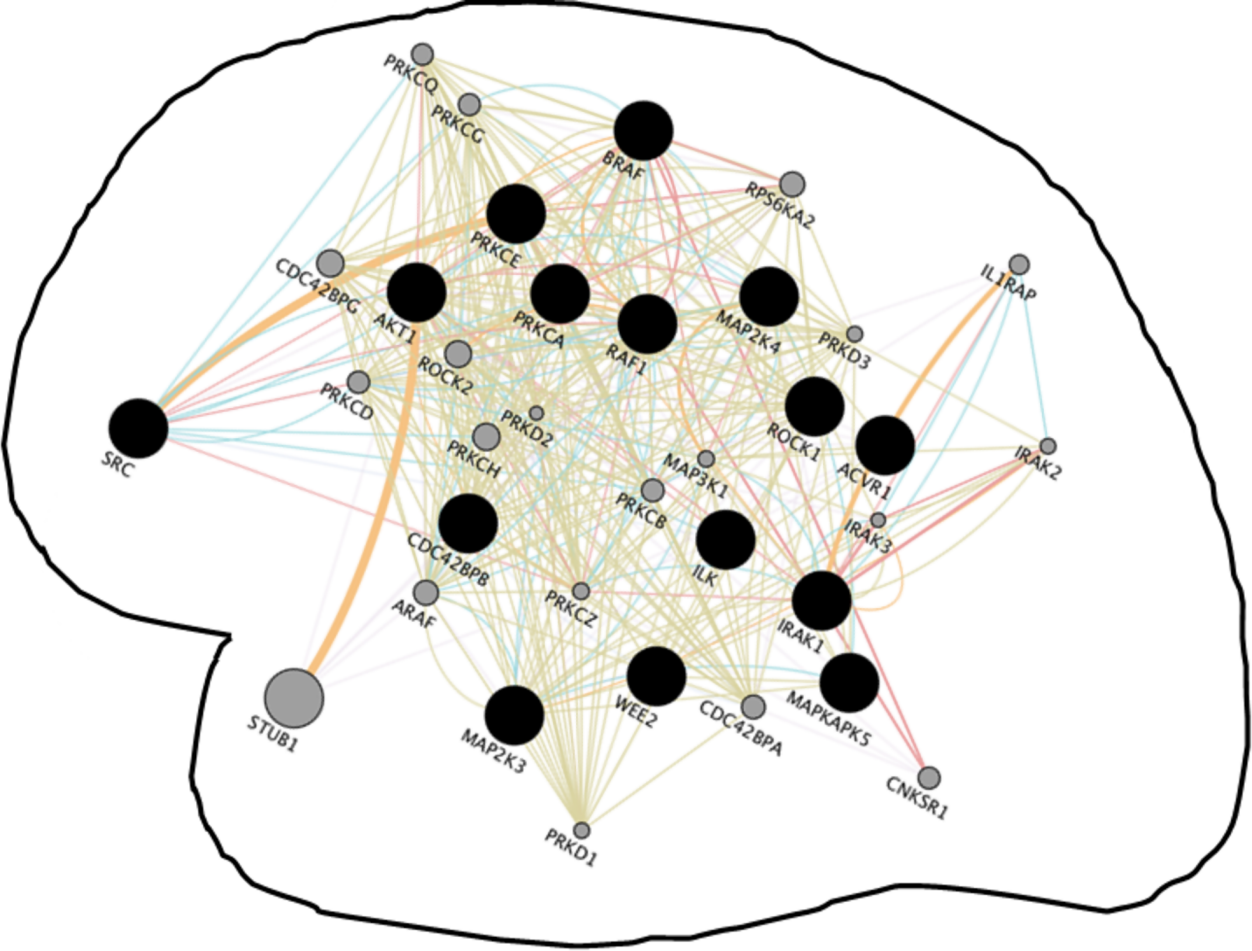
Convergent neighboring gene network shared by Alzheimer’s disease, Parkinson’s disease, Multiple Sclerosis, Age Macular Degeneration, Amyotrophic Lateral Sclerosis, Vascular Dementia, and Restless Leg Syndrome. The network revealed the involvement of immunological pathways as retrieved by geneMANIA with P< 10^−10^. Few of these common genes are also seen in other neuro-disorders: Glaucoma (AKT1, ILK, RAF1, & SRC), Autism-Autism Spectrum (RAF1 & BRAF), Creutzfeldt-Jakob disease (ROCK1 & PRKCE), and Migraine (AKT1). Narcolepsy did not share genes with these common gene sets suggesting possibility unrelated immunopathological mechanisms. Genes in grey dots represent networking genes retrieved by geneMANIA where smaller to higher grey circle sizes represents weaker to the stronger degree of connectivity/associations between two proteins. Colored connecting line between two genes/proteins indicates interactions between them (red-physical; orange-predicated, and green-genetic) and blue lines are part of pathways. Pathways involved here suggest the similar immunological basis of different neuropathogenesis.

Common gene-network signatures of HIV infection and neurological disorders (Alzheimer’s disease, Parkinson’s disease, multiple sclerosis, and age macular degeneration) were compared with a unique convergent gene network shared by seven major neurological disorders. This common network of several neurological disorders was discovered by venn-analyzing neighboring networks of Alzheimer’s disease, Parkinson’s disease, Multiple Sclerosis, Age Macular Degeneration, Amyotrophic Lateral Sclerosis, Vascular Dementia, and Restless Leg Syndrome (S3 Data; Fig 4). A total of 15 common genes were discovered in the network of seven neurological disorders which were used for network development using Cytoscape’s geneMANIA plug-in. The network involved a total of 35 genes which were associated with pathways related to Protein serine/threonine kinase activity, immune response-activating signal transduction, toll-like receptor signaling, activation of immune response pattern recognition receptor signaling, regulation of NF-kappaB, transcription factor activity, platelet activation, etc. Few of these common genes are also seen in other neuro-disorders, for example, AKT1, ILK, RAF1, and SRC gene was common to Glaucoma, Autism-Autism Spectrum included RAF1 and BRAF, Creutzfeldt-Jakob disease included ROCK1 and PRKCE, and Migraine has AKT1. Narcolepsy did not share any gene(s) with these common gene sets.

## Discussion

Existing categorizations of neuroAIDS depend on the preponderance of specific symptoms in neuroAIDS patients which have little or no relevance to the pathological hallmarks. In fact, neuroAIDS constitutes multifaceted symptoms and there are no clear distinctions between its different categories [51]. Moreover, neuroAIDS shows overlapping symptoms and abnormalities with other diseases. For example, dementia, seizures, mood disorders, neuropathic pain, epilepsy, myelopathy, cognitive motor impairment, etc. are common to neuro-disorders such as Alzheimer’s Disease, Parkinson’s Disease, vascular dementia, etc. [51]. This insinuates towards dysregulation of molecular processes which are common to other neuro-disorders. Now, the challenge is to dissect the molecular networks and dysregulated pathways shared between neuroAIDS and different neurological disorders. Some previous studies have identified involvement of few common genes between neuroAIDS and Alzheimer’s, Parkinson’s disease, etc. [44,45]. Here, to best of our knowledge, for the first time, we have analyzed the pathologic-specific genomic association between HIV infection and major neurological disorders. We utilized an integrated network approach based on “random walk algorithm with restart” to quantitatively prioritize genes. This prioritization is performed according to the topological distance and functional relatedness with known genes of other diseases in the functional linkage network (FLN) [46,47]. The basis of FLN ranking of potential disease-related genes utilizes the functional relatedness of those gene(s) which directly or indirectly contributes to a particular disease phenotype. Genetic associations that confer risk to HIV infection and twelve major neurological disorders were retrieved and selected from the Genome-Wide Associations Studies (GWAS) catalog. Although a number of at-risk genes varied among different diseases, these counts did not influence the number of shared-neighboring genes between a specific disease and HIV infection. For example, with only one at-risk gene of vascular dementia, the shared-neighboring genes between vascular dementia and HIV infection were higher than those between HIV and other diseases with more number of at-risk genes (S1 Table). The higher or lower number of shared-neighboring genes may reflect how close or distant a specific disease phenotype may appear during the neuroAIDS condition. Functional analysis of the network of shared-neighboring genes shows the involvement of several important functional pathways (S2 Data). This suggests neuroAIDS involvement in dysregulation of those basic neuronal metabolic machineries which are common to various neuro-disorders. Moreover, detail assessment of network topology reveal interactions of shared genes with at-risk genes (genetic loci) associated with both HIV infection and neurological disorders (Fig 1 & S2 Data). Several studies have implicated same genes or mutations in more than one neuro-disorder. In fact, these kinds of observations are reminiscent for autoimmune disorders. This genetic commonality (close association) may potentiate susceptibility of other neurological disorders and their phenotypic signature may have a reflection in clinical symptoms from the onset of neuroAIDS. In fact, studies have shown that copy number variants and differences in the temporal expression of a gene associated with a neurological disorder can put at risk of others [52].

Large-scale genetic network comparison involving multiples diseases of similar nature may unearth common genetic risk factors associated with a range of neurological illness. The venn analysis of shared-neighboring genes revealed a common set of 10 genes among HIV infection Alzheimer’s disease, Parkinson’s disease, multiple sclerosis, age macular degeneration (Fig 2: EGFR, AKT1, GRB2, MAPK3, IKBKB, CD40, CD14, IRAK1, CD3E, and SYT1). Unlike Mendelian neuro-disorder, these four diseases are sporadic in nature where a slow progressive clinical course and neuronal loss with regional CNS specificity is acquired with their onset [3,52]. Even they feature similar mechanistic characteristics such as apoptosis, generation of reactive oxygen species, generation of proinflammatory factors, ionic channel modulations, and synaptodendritic injury. Sporadic neurodegenerative diseases generally involve modulation of multiple genetic factors at their molecular level [3]. Common genes discovered in this study have also been shown to play a significant role in the pathogenesis of these diseases. EGFR signaling plays a significant role in maintaining neuro-metabolic activities related to neurogenesis and neuron survival. Regulation of EGFR endocytosis via parkin protein drives Parkinson’s pathology [53]. In the case of Alzheimer’s disease, EGFR targeting has been proposed to treat amyloid-β induced memory loss [54]. Increased EGFR expression in cell bodies of astrocytes during multiple sclerosis suggests their role in pathological plaques [55]. The AKT signaling constitutes a significant part of major molecular pathways and has multifaceted role including neuroprotection, cell proliferation, and apoptosis inhibition. Inhibition of Akt signaling associated pathways can be a target for reducing age-related macular degeneration [56] Alzheimer’s disease [57], and Parkinson’s disease [58]. The PI3K/Akt signaling pathway is also involved in mediating an inflammatory response in multiple sclerosis [59]. GRB2 promotes autophagic removal of amyloid-β precursor overload and reduces Alzheimer’s disease-like pathology in neuronal cells [60]. Other proteins of the common network, MAPK3, CD40, CD14, IRAK1, IKBKB, and SYT1 drives different neuropathology in one or other way and they have been discussed below. The role of several proteins such as CD3E from our common network is yet to be defined in these diseases. In fact, genomic signatures of common sporadic forms largely remain unknown [3]. Considering association of common genes in driving different pathologies, we believe a network of these 10 genes may serve as a common canonical microcircuit to establish a link on their pathobiology. Gene-network signature based on these common genes suggests modulations of several pathways such as immune response-activating signal transduction, toll-like receptor signaling, activation of immune response pattern recognition receptor signaling, MyD88-dependent signaling, etc.

The relevance of common gene circuit was further interrogated via mRNA microarray analysis in HIV infection model. Unlike active HIV infection, the latent infection persists for long period and serves as a long-lasting reservoir of rebound viremia. Subpopulations of various cell types have been reported to maintain HIV latency. Cells of monocyte-macrophage lineage can migrate to and from periphery to brain or vice-versa and infect immune-privileged central nervous system during HIV infection. In fact, HIV-infected monocytes/macrophages from the periphery are a major factor in infecting CNS and subsequently neuroAIDS progress which is aggravated with time by active and/or latent infection of macrophages-originated microglia cells and other brain cell types [8,15,17]. Monocytes/macrophages may be considered as stepping stone for neuroAIDS condition and examination of molecular changes in these cells during HIV infection may reveal an inkling of neuroAIDS inception. Significant changes in the expression of 17 out of 29 genes of the bioinformatically discovered common network are found in active and/or latent HIV-infected monocytes (Fig 3). This included the significant change in the expression of 4 at-risk genes of HIV infection (PARD3B, HCP5, HLA-B, and MICB). Neighboring gene circuit may vary depending on presence or absence of one or more dysregulated at-risk genes. At-risk genes in GWAS are based on the different population of patients where each population shows only one (in some cases more than one) at-risk gene dysregulation. Modulations of even a single at-risk gene may be sufficient for infection vulnerability in a specific patients or populations. In the same line, differential expression of specific genes (not all) from the shared network, in our monocytes based in vitro study, may be a reflection of cell-specific reaction to HIV infection where only one or few HIV-specific at-risk genes may get dysregulated. Nonetheless, possible dysregulation of all HIV-specific at-risk genes in different cell types—taken together in a body- cannot be neglected. Different cell types of a patient may include dysregulation of all HIV-specific at-risk genes from GWAS and all common neighboring genes as well. This may be revealed by extending this preliminary study to the other cell types from periphery and brain region. Differentially expressed genes in monocytes, from our common network, have been correlated with different neuronal injury as exampled next. Mutations in SYT1 gene, which binds to SNARE complex, perturb synaptic vesicle cycling [61]. A point mutation in PTPRC gene is strongly associated with multiple sclerosis in the German population [62]. IRAK1 is one of the major genes that drive postnatal nervous system development and is differentially regulated in Alzheimer’s disease and in stressed human astroglial cells [63,64]. Gene AP1B1 has been correlated with the neural-regulated micro-exon in autism spectrum disorder [65]. EGFR is related to oligodendrocyte specification [66] and is a well-established gene for treating neurological disorders. Blocking of EGFR attenuates astrogliosis and protects against ischemic brain injury [67]. Altered expression of CD180 has been reported in peripheral blood leukocytes of Parkinson’s patients [68] and it is significantly dysregulated during cortical-striatal circuit dysfunction [69]. Mutation in IKBKG gene is closely related to CNS anomalies in incontinentia pigmenti patients [70] and Alzheimer’s disease [71]. The role of MAPK pathways is shown in neurodegenerative diseases such as Alzheimer’s disease, Parkinson’s disease, and amyotrophic lateral sclerosis [72]. The CD40 promotes macrophages/microglia-induced inflammatory response and its interaction with CD154 enhances neurotoxins secretion [73]. Deletion of CD14 attenuates Alzheimer’s disease pathology [74] and polymorphism in a CD14 monocyte receptor is a genetic risk factor for Parkinson’s disease [75]. The PELI2 gene is differentially expressed in human neural progenitor cells containing overexpressed MEF-2 transcriptional factor which is a key determinant in synaptic plasticity and neuronal survival [76]. Also, PELI2 is one of the conserved targets of a brain-enriched microRNA, miRNA-128 which is differentially expressed in brain tumors and neuronal differentiation [77]. The CDKN1B (p27) regulates oligodendrocyte differentiation [78] and neurogenesis in the adult subventricular zone [79]. Interaction of CDKN1B with HIV Tat in driving neurotoxicity has been shown earlier [80,81]. The RPS6KA2 gene can influence brain network related to attention performance [82] and in glioma tumor formation [83]. Similarly, other genes of the common gene-circuits (Fig 2) regulate different aspects of neuronal regulations in one or other way. Once again, aforementioned correlation of altered genes expression during HIV infection should be seen as a reflection of complex neuroAIDS condition where characteristics of various neuronal pathologies appear. Therefore, the bioinformatically discovered common pathway in our study may have remarkable relevance in driving neuroAIDS condition. Nonetheless, an in vitro condition represents only a short HIV infection period in very specific artificial circumstances which can be little compared to the complex physiological networks in the body. The relevance of network discovered in our study should be considered cautiously while comparing with an infected subject. This should only be seen in a context to provide a prospective platform that how gene-circuits belonging to other neuro-disorders may be convoluted during real-time neuroAIDS condition. Again, an extension of this study to different cell types and/or population may reveal a more reasonable representation to be emulated for the real-time condition.

Moreover, we discovered a novel shared gene-network among neurological disorders (excluding HIV infection). Venn analysis of neighboring interactants of Alzheimer’s disease, Parkinson’s disease, Multiple Sclerosis, Age Macular Degeneration, Amyotrophic Lateral Sclerosis, Vascular Dementia, and Restless Leg Syndrome revealed a common set of 15 genes among these disorders (S2 Data and Fig 3). The visual network of these common genes showed the involvement of various innate immunity pathways suggesting commonalities in the immunological basis of different neuropathogenesis (Figs 2 & 3). Nonetheless, this network possesses little molecular crossover with the HIV infection-specific shared gene-circuit. This insinuates towards a different neuro-immunological molecular mechanism of neuroAIDS in compare to other neurological disorders. It is possible that difference in the expression of these marker genes and their genomic network(s) may unravel the common mechanism of sporadic neuro-disorders. Nonetheless, this can only represent a small subset of the network when the arrangement of billions of networks are dictated on the basis of neuronal cell morphology, electrophysiological properties, and choreography of the biosynthesis, trafficking and molecular interactions and connectivity [84]. Non-specific effects of disease-causing genomic mutations are putatively believed to be key driving factors of neuro-disorders and as such genes in common networks may provide a clue for additional risk factors. However, the in silico analysis based on publically available database possess an important caveat in their technical noise associated with the accuracy of clinical annotations. This is due to the variable quality of measurements in the different data source. Notably, this makes comparisons across different data sets challenging and any discovery solely based on in silico approach is unlikely to have clinical acceptance [85]. Exploratory follow-ups of these in silico findings—involving clinical resolution—may substantiate the gene functional similarities between the diseases. It is possible that quantitative variations of common genes set, depending upon individual genetic background, may drive either the presence or absence of specific neuro-disorders type. Prevailing inter-individual variability in neuroAIDS conditions among HIV patients–which is also a major confounding factor in the utility of in silico analysis–can be decoded by knowing if and when aspects of “on-and-off” switching of specific identified genes. This will require a systematic proof-of-concept study by first-hand verification of in silico findings in primary cells from uninfected and/or from HIV-infected individuals. Such study may reveal presence or absence of identified genes and effects of donor variation on gene targets in real time infection scenario. An extension of our analysis based on different peripheral and neuronal cell lines from infected individuals will be able to provide a wider screen to correlate progression of neuroAIDS to other neurological diseases. Furthermore, since the majority of genes from our discovered pathways target various inflammatory effects, a calculated effort towards immunosuppressive therapies (based on common genes) may prove critical for treatment of neuroAIDS and/or other neuropathologies.

In summary, the HAART treatment regimen has increased the longevity of HIV patients in recent years; nonetheless, it is ineffective on checking neuroAIDS which, in turn, has shaped up as a global problem. Currently, specific diagnosis tools or protocols for this epidemic is lacking in entirety. The classification of gene-network signatures of functionally associated neuropathology will dissect the multifaceted nature of the neuroAIDS condition and will provide a platform to prioritize genetic biomarkers and drug targets. In fact, the geno-bioinformatics analysis in this post-genomic era possesses unrealized opportunities to delineate molecular mechanisms of several neuro-disorders which are not feasible via conventional neurological examination. Such potential for neurodegenerative disorders is even higher because they show similar lesions and as such clues to their origin may be found in common genes circuit.

## Ethics approval and consent to participate

As this study did not involve any animal or human participants, human data or human tissue, ethical committee approval is not required.

## Supporting information

S1 Fig Control p24 assay. A control experiment was performed to determine presence of latent HIV in U1 cells. As such U1 cells were activated by PMS (10 nM) for 4 hr, washed with PBS, and cultured for 5 days. The p24 quantification study in culture supernatant suggests original U1 cells are latent because exposure of PMA resulted in very high load of p24 antigens.
(DOCX)

S2 Fig Control p24 assay. A control experiment was performed to determine initiation of active infection in U 937 cells. As such U937 cells were activated by PMS (10 nM) for 4 hr, washed with PBS, and infected with HIV for 5 days. The p24 quantification study in culture supernatant suggests active HIV infection in U937 cells.
(DOCX)

S1 Table Number of genes retrieved from the GWAS catalog for generation of neighboring genes. Venn analysis of neighboring genes between AIDS an each neurological disorders revealed their shared genes.
(DOCX)

S1 Data Shared neighbouring genes. Shared neighboring genes between AIDS and different neurological disorders.
(XLSX)

S2 Data Pathway list. Visual networks of shared genes of AIDS and different neurological networks reveales involvement of several important functional pathways.
(XLSX)

S3 Data Neighbouring genes of neurological disorders. Common genes from gene interactants of different neurological disorders are represented.
(XLSX)

S4 Data At-risk genes of HIV and different neurological disorders. At-risk alleles of AIDS and twelve major neurological disorders from GWAS catalog have been listed.
(XLSX)
