# Supplementary material for: Common gene-network signature of different neurological disorders and their potential implications to neuroAIDS
Source: PLoS One. 2017 Aug 8;12(8):e0181642. doi: 10.1371/journal.pone.0181642 (PMC5549695; doi:10.1371/journal.pone.0181642)

**S1 Fig:**

A control experiment was performed to determine presence of latent HIV in U1 cells. As such U1 cells were activated by PMS (10 nM) for 4 hr, washed with PBS, and cultured for 5 days. The p24 quantification study in culture supernatant suggests original U1 cells are latent because exposure of PMA resulted in very high load of p24 antigens.


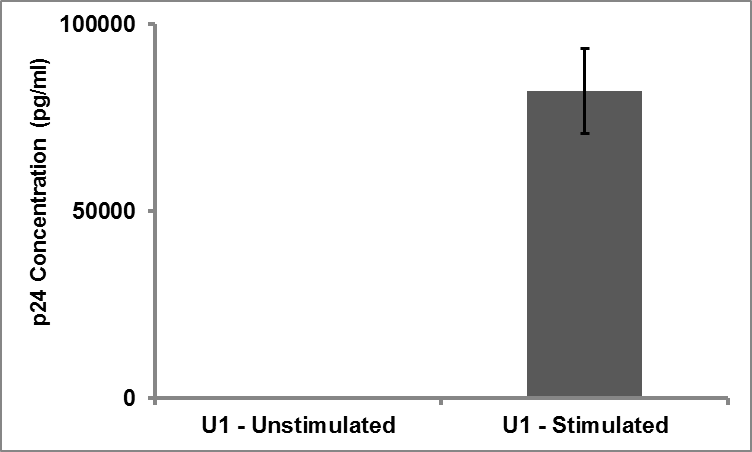

Supplement: S1 Fig — A control experiment was performed to determine presence of latent HIV in U1 cells. As such U1 cells were activated by PMS (10 nM) for 4 hr, washed with PBS, and cultured for 5 days. The p24 quantification study in culture supernatant suggests original U1 cells are latent because exposure of PMA resulted in very high load of p24 antigens. (DOCX) [file pone.0181642.s001.docx]
