# Supplementary material for: Common gene-network signature of different neurological disorders and their potential implications to neuroAIDS
Source: PLoS One. 2017 Aug 8;12(8):e0181642. doi: 10.1371/journal.pone.0181642 (PMC5549695; doi:10.1371/journal.pone.0181642)

**S2 Fig**:

A control experiment was performed to determine initiation of active infection in U 937 cells. As such U937 cells were activated by PMS (10 nM) for 4 hr, washed with PBS, and infected with HIV for 5 days. The p24 quantification study in culture supernatant suggests active HIV infection in U937 cells.


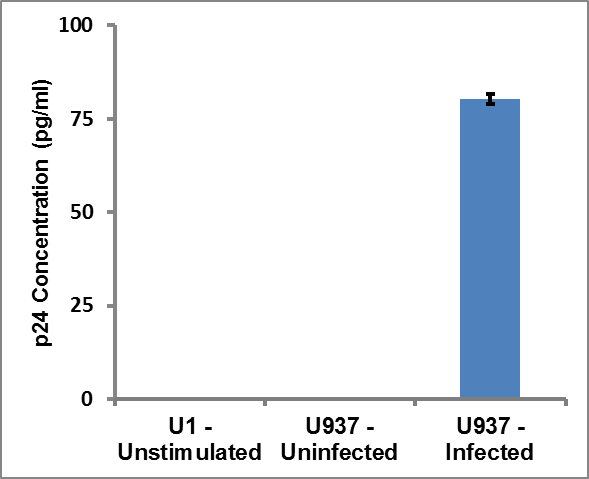

Supplement: S2 Fig — A control experiment was performed to determine initiation of active infection in U 937 cells. As such U937 cells were activated by PMS (10 nM) for 4 hr, washed with PBS, and infected with HIV for 5 days. The p24 quantification study in culture supernatant suggests active HIV infection in U937 cells. (DOCX) [file pone.0181642.s002.docx]
