# Supplementary material for: Common gene-network signature of different neurological disorders and their potential implications to neuroAIDS
Source: PLoS One. 2017 Aug 8;12(8):e0181642. doi: 10.1371/journal.pone.0181642 (PMC5549695; doi:10.1371/journal.pone.0181642)
Supplement: S1 Table — Venn analysis of neighboring genes between AIDS an each neurological disorders revealed their shared genes. (DOCX) [file pone.0181642.s003.docx]

**S1 Table**:

Number of genes retrieved from the Genome-Wide Associations Studies (GWAS) catalog for generation of neighboring genes. Venn analysis of neighboring genes between AIDS an each neurological disorders revealed their shared genes.

| **Disease** | **AIDS** | **Amyotrophic**  **Lateral Sclerosis** | **Age macular**  **Degeneration** | **Alzheimer’s**  **Disease** | **Parkinson’s**  **Disease** | **Glaucoma** | **Restless**  **Leg Syndrome** | **Vascular Dementia** | **Creutzfeldt-Jakob**  **Disease** | **Migraine** | **Narcolepsy** | **Autism-Autism Spectrum** | **Multiple sclerosis** |
| --- | --- | --- | --- | --- | --- | --- | --- | --- | --- | --- | --- | --- | --- |
| **At-risk genes** | 9 | 15 | 45 | 71 | 30 | 20 | 9 | 1 | 3 | 20 | 8 | 27 | 88 |
| **Neighboring genes** | 785 | 591 | 785 | 1257 | 921 | 418 | 326 | 228 | 159 | 336 | 92 | 482 | 1982 |
| **Shared genes with AIDS** | _ | 58 | 248 | 199 | 102 | 45 | 26 | 54 | 15 | 61 | 42 | 110 | 387 |
